# Supplementary material for: Cardiovascular disease risk profile and management practices in 45 low-income and middle-income countries: A cross-sectional study of nationally representative individual-level survey data
Source: PLoS Med. 2021 Mar 4;18(3):e1003485. doi: 10.1371/journal.pmed.1003485 (PMC7932723; doi:10.1371/journal.pmed.1003485)
Supplement: S1 Fig — (DOCX) [file pmed.1003485.s002.docx]

## Percent with missing data for CVD risk estimation by country*


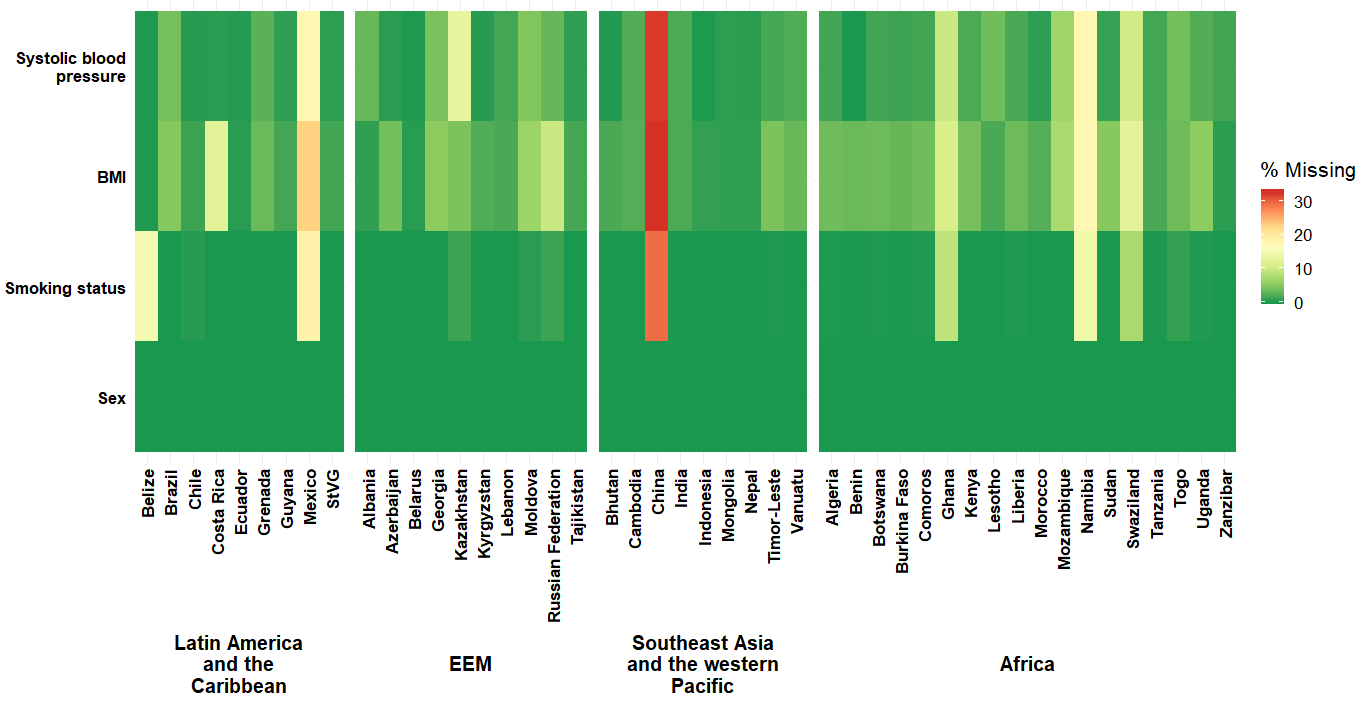


*EEM: Europe and the eastern Mediterranean; StVG: Saint Vincent and the Grenadines
