# Supplementary material for: Cardiovascular disease risk profile and management practices in 45 low-income and middle-income countries: A cross-sectional study of nationally representative individual-level survey data
Source: PLoS Med. 2021 Mar 4;18(3):e1003485. doi: 10.1371/journal.pmed.1003485 (PMC7932723; doi:10.1371/journal.pmed.1003485)
Supplement: S2 Fig — (DOCX) [file pmed.1003485.s003.docx]

## CVD risk profile by country for 40-64 year old men and women^¶^


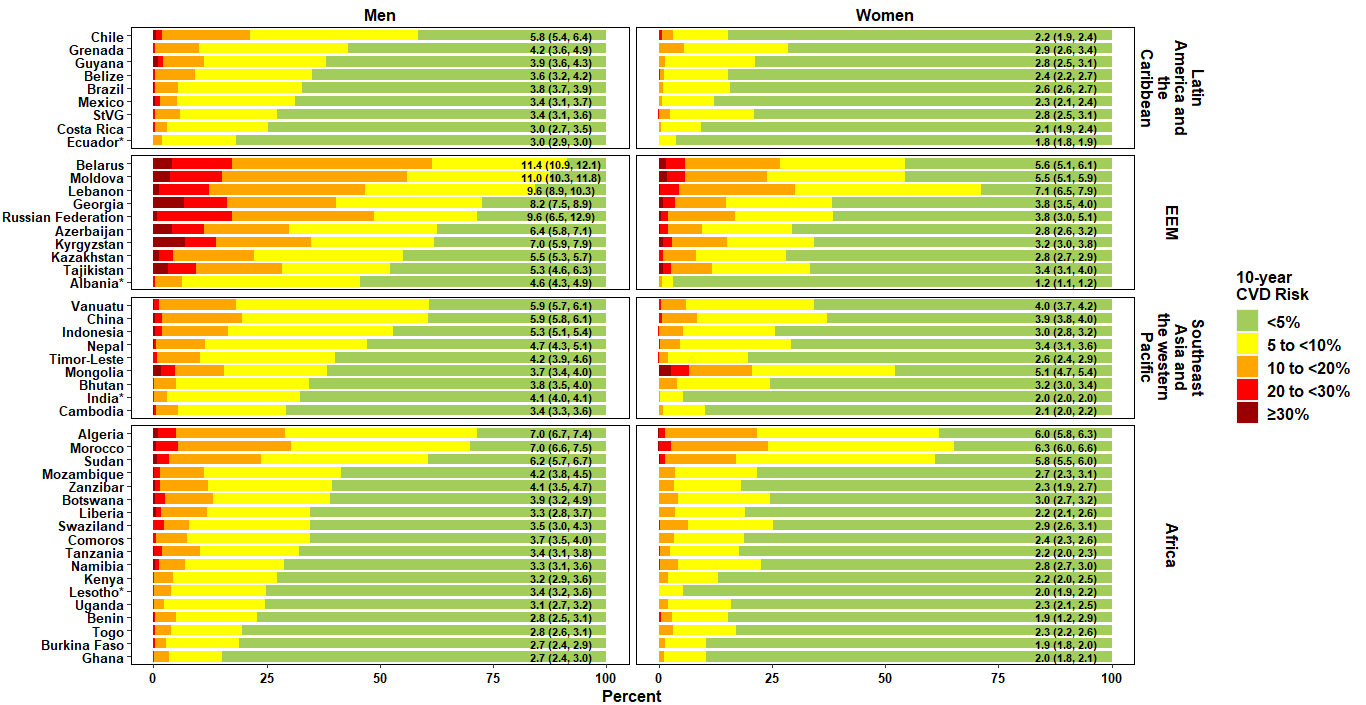


^¶^ The numbers in the green section of the stacked bars represent the median 10-year CVD risk along with 95% confidence intervals for the country. All estimates are age-standardized using the Global Burden of Disease project’s 2017 global population.

* The age range included in the sample varied in these four countries: India – 40-49 years for women and 40-54 years for men; Ecquador – 40-59 years for both men and women; Lesotho – 40-49 years for women and 40-59 years for men; Albania – 40-49 years for both men and women.

Abbreviations: EEM: Europe and the eastern Mediterranean; StVG: Saint Vincent and the Grenadines.
