# Supplementary material for: Cardiovascular disease risk profile and management practices in 45 low-income and middle-income countries: A cross-sectional study of nationally representative individual-level survey data
Source: PLoS Med. 2021 Mar 4;18(3):e1003485. doi: 10.1371/journal.pmed.1003485 (PMC7932723; doi:10.1371/journal.pmed.1003485)
Supplement: S4 Fig — (DOCX) [file pmed.1003485.s005.docx]

## Relative change (%) in 10-year CVD risk for males by educational level, household wealth, marital status and employment status^¶^

|  | Primary school or higher education vs. no formal education | Upper three wealth quintiles vs. bottom two quintiles | Married/cohabiting vs. non-married | Working in past12 months vs. not working |
| --- | --- | --- | --- | --- |
| 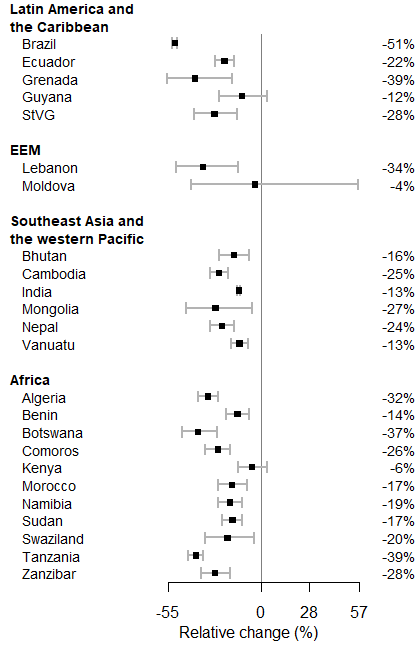 | 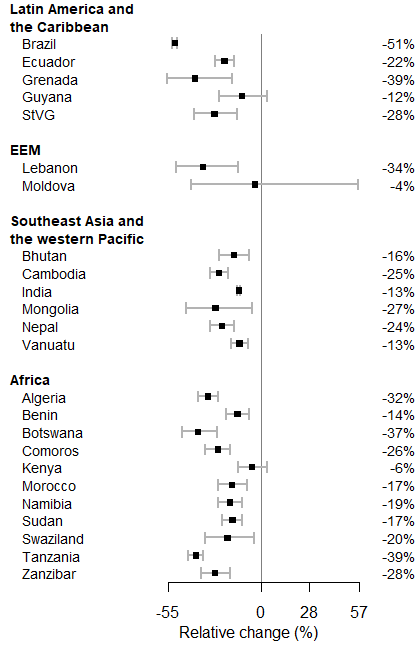 | 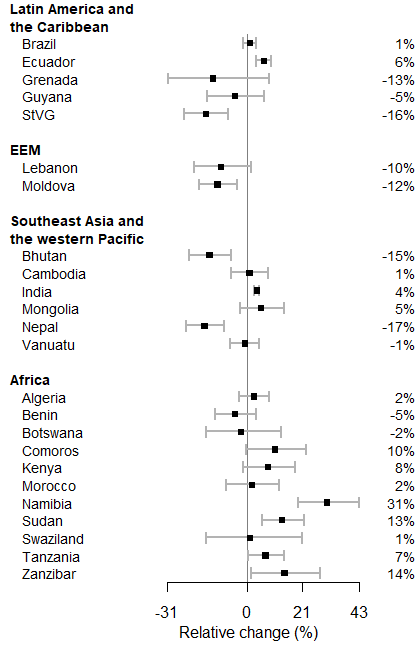 | 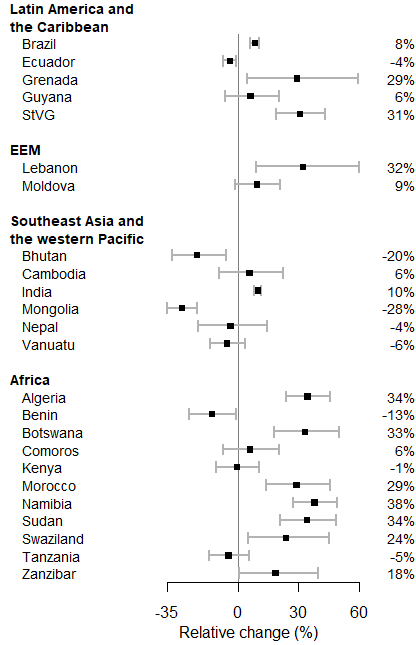 | 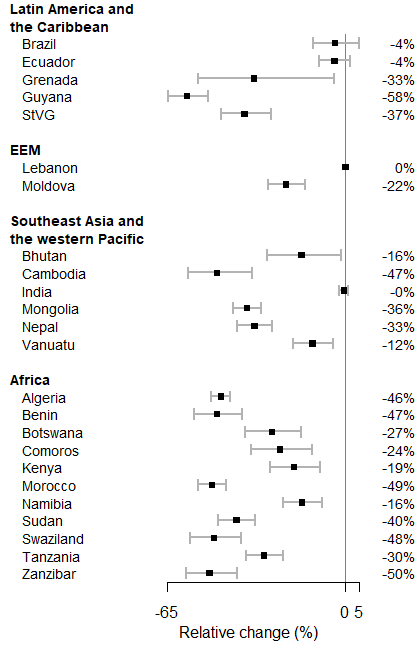 |

^¶^ These are estimates from linear mixed models with the primary sampling units as the clusters. The outcome is logarithm of CVD risk and the predictors are gender, educational level (High school or above vs. primary school/no schooling) household wealth quintile (middle/richer/richest vs. poorer/poorest), marital status (married/cohabiting vs. never married/separated/divorced/widowed) and employment status in the last 12 months (working vs. not working). The estimates for Grenada and Morocco are based on linear regression as there are no primary sampling units and a single participant was sampled from each household. The countries with estimates not plotted either had a predictor missing or there were less than 5 participants in a category for one or more predictorsAbbreviations: EEM: Europe and the eastern Mediterranean; StVG: Saint Vincent and the Grenadines.
