# Supplementary material for: Cardiovascular disease risk profile and management practices in 45 low-income and middle-income countries: A cross-sectional study of nationally representative individual-level survey data
Source: PLoS Med. 2021 Mar 4;18(3):e1003485. doi: 10.1371/journal.pmed.1003485 (PMC7932723; doi:10.1371/journal.pmed.1003485)
Supplement: S6 Fig — (DOCX) [file pmed.1003485.s007.docx]

## Risk ratios (%) for taking BP medication by educational level, household wealth, marital status and employment status, for individuals not indicated for medication per WHO/ISH guidelines.

|  | Primary school or higher education | Upper three wealth quintiles | Married/cohabiting | Working in past12 months |
| --- | --- | --- | --- | --- |
| 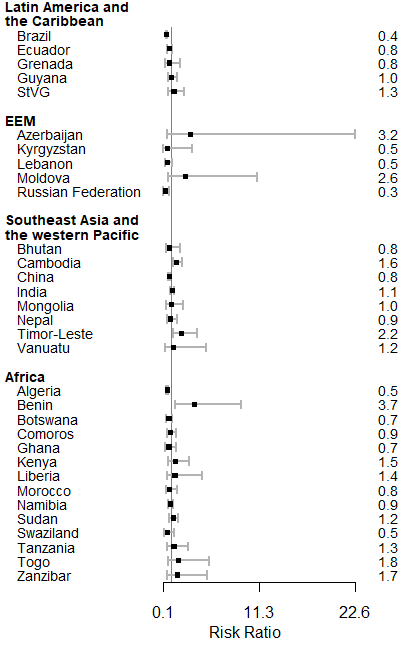 | 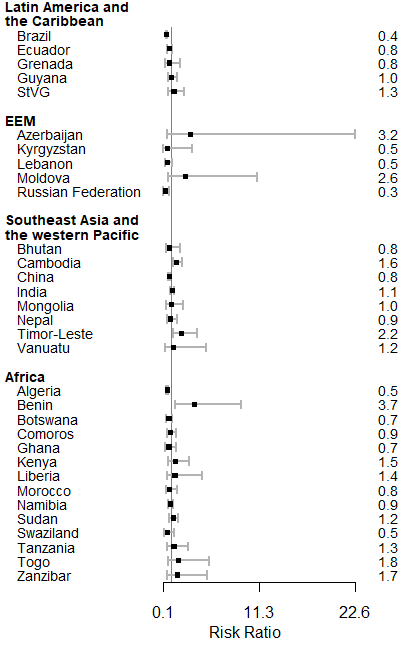 | 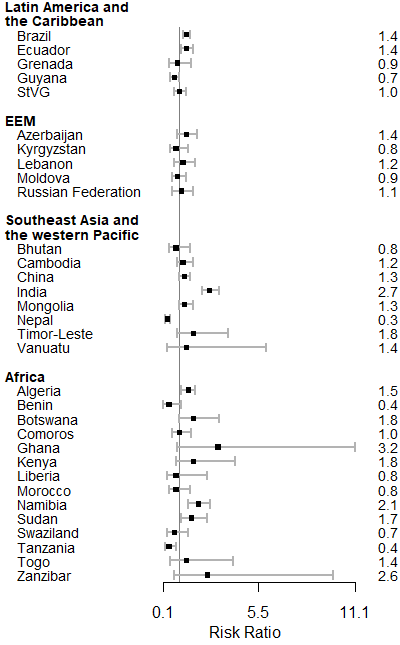 | 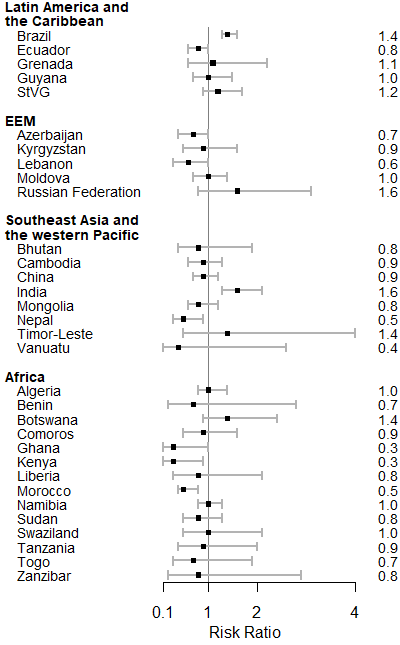 | 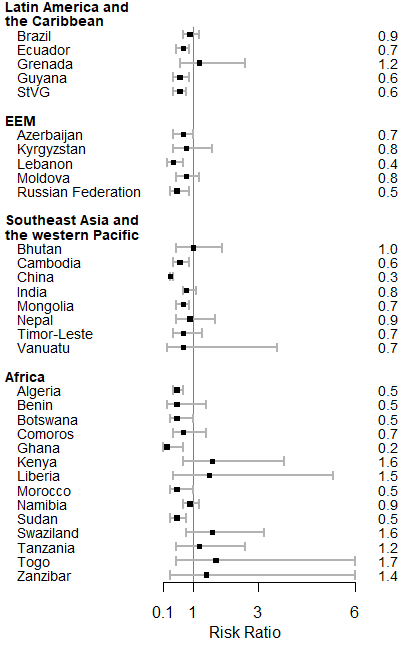 |
