# Supplementary material for: Cardiovascular disease risk profile and management practices in 45 low-income and middle-income countries: A cross-sectional study of nationally representative individual-level survey data
Source: PLoS Med. 2021 Mar 4;18(3):e1003485. doi: 10.1371/journal.pmed.1003485 (PMC7932723; doi:10.1371/journal.pmed.1003485)
Supplement: S1 Table — (DOCX) [file pmed.1003485.s008.docx]

## Summary of surveys included

| **Country** | **Source** | **Year** |
| --- | --- | --- |
| Albania | Demographic and Health Survey | 2008 |
| Algeria | STEPS | 2016 |
| Azerbaijan | STEPS | 2017 |
| Belarus | STEPS | 2016 |
| Belize | Central America Diabetes Initiative | 2005-6 |
| Benin | STEPS | 2015 |
| Bhutan | STEPS | 2014 |
| Botswana | STEPS | 2014 |
| Brazil | Pesquisa Nacional de Saude(PNC) | 2013 |
| Burkina Faso | STEPS | 2013 |
| Cambodia | STEPS | 2010 |
| Chile | National Health Survey | 2009-10 |
| China | China Health and Nutrition Survey | 2009 |
| Comoros | STEPS | 2011 |
| Costa Rica | STEPS | 2010 |
| Ecuador | Ecquador National Health and Nutrition Survey | 2012 |
| Georgia | STEPS | 2016 |
| Ghana | SAGE | 2007/8 |
| Grenada | STEPS | 2009-11 |
| Guyana | STEPS | 2016 |
| India | Demographic and Health Survey | 2015-2016 |
| Indonesia | Indonesian Family Life Survey | 2014 |
| Kazakhstan | Household Health Survey | 2012 |
| Kenya | STEPS | 2015 |
| Kyrgyzstan | STEPS | 2013 |
| Lebanon | STEPS | 2008-09 |
| Lesotho | Demographic and Health Survey | 2014 |
| Liberia | STEPS | 2011 |
| Mexico | Mexican Family Life Survey | 2009-12 |
| Moldova | STEPS | 2013 |
| Mongolia | STEPS | 2009 |
| Morocco | STEPS | 2017 |
| Mozambique | STEPS | 2005 |
| Namibia | Demographic and Health Survey | 2013 |
| Nepal | STEPS | 2013 |
| Russian Federation | Study on Global Ageing and Adult health | 2007/8 |
| St. Vincent & the Grenadines | STEPS | 2013 |
| Sudan | STEPS | 2016 |
| Swaziland | STEPS | 2014 |
| Tajikistan | STEPS | 2016 |
| Tanzania | STEPS | 2012 |
| Timor Leste | STEPS | 2014 |
| Togo | STEPS | 2010 |
| Uganda | STEPS | 2014 |
| Vanuatu | STEPS | 2011 |
| Zanzibar | STEPS | 2011 |
