# Supplementary material for: Cardiovascular disease risk profile and management practices in 45 low-income and middle-income countries: A cross-sectional study of nationally representative individual-level survey data
Source: PLoS Med. 2021 Mar 4;18(3):e1003485. doi: 10.1371/journal.pmed.1003485 (PMC7932723; doi:10.1371/journal.pmed.1003485)
Supplement: S2 Table — (DOCX) [file pmed.1003485.s009.docx]

## Sample characteristics among those excluded due to a missing outcome variable.

|  | **Missing outcome variable^¥^**  **n=** **25,752** | **Included in analysis**  **n=600,484** |
| --- | --- | --- |
| Characteristic | Percent^*^ | Percent^*^ |
| Sex |  |  |
| Male | 37.2 | 47.4 |
| Female | 62.8 | 52.6 |
| Missing | 0.0 | 0.0 |
| Age |  |  |
| 30-34 years | 25.7 | 19.2 |
| 35-44 years | 34.6 | 34.9 |
| 45-54 years | 19.4 | 25.2 |
| 55-64 years | 14.6 | 16.0 |
| 65+ years | 5.8 | 4.8 |
| Missing | 0.0 | 0.0 |
| Current smoker |  |  |
| No | 74.5 | 80.4 |
| Yes | 15.5 | 19.6 |
| Missing | 10.0 | 0.0 |
| Educational Attainment |  |  |
| Less than primary school | 29.6 | 30.3 |
| Primary school or higher | 69.8 | 69.6 |
| Missing | 0.5 | 0.1 |
| Household Wealth Quintile |  |  |
| 1 | 14.5 | 14.5 |
| 2 | 12.5 | 14.8 |
| 3 | 13.1 | 14.7 |
| 4 | 13.1 | 14.8 |
| 5 | 16.5 | 15.6 |
| Missing | 30.3 | 25.7 |

*Values are weighted with each country having the same weight regardless of population and sample size.

^¥^ Participants missing one or more predictors in World Health Organization cardiovascular disease risk charts — age, sex, current smoking status, body mass index, and systolic blood pressure.
