# Supplementary material for: Cardiovascular disease risk profile and management practices in 45 low-income and middle-income countries: A cross-sectional study of nationally representative individual-level survey data
Source: PLoS Med. 2021 Mar 4;18(3):e1003485. doi: 10.1371/journal.pmed.1003485 (PMC7932723; doi:10.1371/journal.pmed.1003485)
Supplement: S3 Table — (DOCX) [file pmed.1003485.s010.docx]

## Risk factor distribution by educational level, household wealth, marital status and employment status, among males and females across the four regions

|  |  |  | **Females** | | | |  | **Males** | | | |
| --- | --- | --- | --- | --- | --- | --- | --- | --- | --- | --- | --- |
| **SES indicator** | **Region** | **Indicator level** | **Median age** | **Median current smoker %** | **Median BMI** | **Median SBP** |  | **Median age** | **Median current smoker %** | **Median BMI** | **Median SBP** |
| Educational attainment | Latin America and the Caribbean | Primary school or higher education | 43.0 | 6.4 | 28.1 | 120 |  | 43.0 | 25.8 | 26.3 | 127 |
|  |  | Less than primary school | 52.8 | 3.0 | 28.4 | 126 |  | 50.0 | 28.2 | 25.1 | 131 |
|  | EEM | Primary school or higher education | 47.0 | 3.9 | 27.4 | 128 |  | 45.5 | 47.7 | 26.4 | 132 |
|  |  | Less than primary school | 54.5 | 0.0 | 29.1 | 132 |  | 42.9 | 54.1 | 24.7 | 128 |
|  | Southeast Asia and the western Pacific | Primary school or higher education | 39.0 | 4.3 | 24.2 | 124 |  | 41.0 | 47.7 | 23.3 | 124 |
|  |  | Less than primary school | 45.0 | 8.6 | 23.0 | 126 |  | 45.4 | 58.8 | 21.6 | 126 |
|  | Africa | Primary school or higher education | 39.5 | 1.5 | 26.3 | 123 |  | 40.5 | 22.2 | 23.0 | 127 |
|  |  | Less than primary school | 45.0 | 2.7 | 23.3 | 127 |  | 45.0 | 24.5 | 21.8 | 128 |
|  |  |  |  |  |  |  |  |  |  |  |  |
| Household wealth quintile | Latin America and the Caribbean | Non-poor household | 42.0 | 7.2 | 28.0 | 123 |  | 42.5 | 24.6 | 26.1 | 127 |
|  |  | Poor household | 44.0 | 6.6 | 28.2 | 126 |  | 45.3 | 32.5 | 25.0 | 128 |
|  | EEM | Non-poor household | 45.0 | 5.3 | 27.1 | 125 |  | 45.0 | 46.6 | 26.6 | 131 |
|  |  | Poor household | 47.0 | 2.4 | 27.6 | 131 |  | 47.0 | 49.5 | 26.2 | 130 |
|  | Southeast Asia and the western Pacific | Non-poor household | 41.0 | 6.1 | 23.9 | 124 |  | 42.0 | 48.0 | 23.5 | 124 |
|  |  | Poor household | 44.0 | 8.1 | 23.4 | 128 |  | 45.0 | 61.2 | 22.6 | 126 |
|  | Africa | Non-poor household | 40.0 | 1.2 | 26.5 | 126 |  | 41.0 | 20.7 | 23.2 | 128 |
|  |  | Poor household | 41.8 | 2.4 | 23.3 | 124 |  | 42.2 | 25.7 | 21.9 | 127 |
|  |  |  |  |  |  |  |  |  |  |  |  |
| Marital status | Latin America and the Caribbean | With a partner | 43.6 | 6.3 | 28.4 | 122 |  | 45.5 | 24.5 | 26.8 | 128 |
|  |  | Without a partner | 45.5 | 7.0 | 28.2 | 124 |  | 42.3 | 33.5 | 25.7 | 126 |
|  | EEM | With a partner | 46.0 | 3.9 | 27.5 | 128 |  | 46.0 | 44.9 | 26.7 | 131 |
|  |  | Without a partner | 52.0 | 7.4 | 27.4 | 131 |  | 40.2 | 52.9 | 25.4 | 128 |
|  | Southeast Asia and the western Pacific | With a partner | 42.0 | 6.7 | 23.3 | 124 |  | 42.0 | 56.1 | 22.8 | 124 |
|  |  | Without a partner | 47.0 | 7.3 | 23.2 | 129 |  | 46.0 | 49.0 | 21.9 | 126 |
|  | Africa | With a partner | 40.0 | 2.0 | 24.8 | 124 |  | 43.0 | 20.8 | 22.8 | 127 |
|  |  | Without a partner | 45.8 | 2.7 | 24.3 | 126 |  | 38.0 | 28.8 | 21.9 | 126 |
|  |  |  |  |  |  |  |  |  |  |  |  |
| Employment status | Latin America and the Caribbean | Working | 43.0 | 5.4 | 28.2 | 120 |  | 42.5 | 27.8 | 26.5 | 127 |
|  |  | Not working | 46.0 | 5.4 | 28.7 | 123 |  | 53.1 | 27.3 | 26.4 | 130 |
|  | EEM | Working | 44.5 | 7.9 | 27.1 | 125 |  | 43.3 | 49.1 | 26.8 | 130 |
|  |  | Not working | 49.0 | 4.9 | 28.1 | 130 |  | 51.0 | 47.4 | 26.5 | 134 |
|  | Southeast Asia and the western Pacific | Working | 41.0 | 8.2 | 23.1 | 123 |  | 41.5 | 49.6 | 23.1 | 124 |
|  |  | Not working | 44.5 | 7.5 | 23.4 | 125 |  | 48.5 | 43.7 | 22.5 | 127 |
|  | Africa | Working | 40.5 | 1.8 | 25.5 | 124 |  | 42.0 | 23.0 | 22.8 | 127 |
|  |  | Not working | 42.0 | 2.7 | 23.8 | 127 |  | 47.0 | 23.6 | 22.8 | 129 |
