# Supplementary material for: Cardiovascular disease risk profile and management practices in 45 low-income and middle-income countries: A cross-sectional study of nationally representative individual-level survey data
Source: PLoS Med. 2021 Mar 4;18(3):e1003485. doi: 10.1371/journal.pmed.1003485 (PMC7932723; doi:10.1371/journal.pmed.1003485)
Supplement: S1 Text — (DOCX) [file pmed.1003485.s011.docx]

## Computation of household wealth quintile

Most World Health Organization STEPS surveys did not collect information on dwelling characteristics or household ownership of durable goods, but instead asked about household income in the past year (with the option to provide an estimate per week, month, or year) and, if unable to provide an estimate, the opportunity to give a range against pre-coded categories. Using both household income as a continuous variable and the pre-coded ranges, we created unweighted household wealth quintiles assuming that national incomes followed a log-normal distribution (whereby we used the procedure developed by Harttgen and Vollmer^1^). If a survey asked about dwelling characteristics and household ownership of durable goods, we computed a household wealth index using the standard approach of the Demographic and Health Surveys.^2,3^ Specifically, a binary indicator was generated for each dwelling characteristic and asset. We then ran a principal component analysis (PCA) on these binary indicator variables and extracted the first (unrotated) principal component. The household wealth measure that was available in each survey is shown in the table below.

| **Wealth measure** | **Country** |  |
| --- | --- | --- |
| Asset index | Albania, Brazil, Ecuador, Ghana, India, Indonesia, Kenya, Namibia, Nepal, Russian Federation |  |
| Continuous income | Bhutan, China, Mexico, Kazakhstan, Timor Leste |  |
| Continuous income and quintiles | Algeria, Azerbaijan, Botswana, Cambodia, Comoros*, Eswatini*, Georgia, Kyrgyzstan, Lesotho, Liberia*, Moldova, Tajikistan, Tanzania, Togo*, Uganda*, Vanuatu, Zanzibar |  |
| Continuous income and categories | Benin, Grenada, Guyana, Lebanon, Mongolia*, Morocco |  |
| Income categories or quintiles only | St, Vincent & the Grenadines, Sudan |  |
| No income | Belarus, Belize, Burkina Faso, Chile, Costa Rica, Mozambique |  |
| *Quintiles were not used as they displayed large discrepancies with respect to continuous income range or could not be correctly identified. | | |

1. Harttgen K, Vollmer S. Using an Asset Index to Simulate Household Income. *Economics Letters.* 2013;121(2):257-262.

2. Rutstein S, Johnson K. *The DHS Wealth Index.* 2004.

3. Filmer D, Pritchett LH. Estimating wealth effects without expenditure data--or tears: an application to educational enrollments in states of India. *Demography.* 2001;38(1):115-132.
