# Supplementary material for: Cardiovascular disease risk profile and management practices in 45 low-income and middle-income countries: A cross-sectional study of nationally representative individual-level survey data
Source: PLoS Med. 2021 Mar 4;18(3):e1003485. doi: 10.1371/journal.pmed.1003485 (PMC7932723; doi:10.1371/journal.pmed.1003485)
Supplement: S2 Text — (DOCX) [file pmed.1003485.s012.docx]

# Statistical Analysis Plan

**Cardiovascular disease risk profile and management gaps in low- and middle-income countries**

**Statistical Analysis Plan**

Version 1.0

9 May 2019

**Authors**

Arpita Ghosh, David Peiris

Table of Contents

[1 Administrative Information 3](#_Toc56083327)

[1.1 Revision history 3](#_Toc56083328)

[1.2 Contributors to the statistical analysis plan 3](#_Toc56083329)

[1.1.1 Roles and responsibilities 3](#_Toc56083330)

[2 Overview 3](#_Toc56083331)

[2.1 Objective 3](#_Toc56083332)

[2.2 Cohort Description 3](#_Toc56083333)

[2.3 Data Sources 4](#_Toc56083334)

[3 Outcomes 5](#_Toc56083335)

[4 Statistical Methods 5](#_Toc56083336)

[5 Results 6](#_Toc56083337)

[6 Amendments 6](#_Toc56083338)

**Administrative Information**

Revision history

| Version | Date | Details |
| --- | --- | --- |
| 1.0 | 9 May 2019 | Approved for submission by the George Institute |

Contributors to the statistical analysis plan

Roles and responsibilities

| Name | Affiliation | Role on study | SAP contribution |
| --- | --- | --- | --- |
| Professor David Peiris | The George Institute for Global Health, UNSW Sydney | Principal Investigator | Prepared initial draft and revisions |
| Dr Arpita Ghosh | The George Institute for Global Health, UNSW Sydney | Study statistician | Prepared initial draft and revisions |

Overview

Chronic diseases including, diabetes and cardiovascular disease (CVD), are now the leading causes of death and disability in low- and middle-income countries (LMICs)1. CVD represents a large and increasing health and economic burden globally. Coordinated action with respect to CVD is especially important for several reasons: (1) CVD occur at younger ages globally when compared to the United States and Europe, and thus have a greater impact on years of healthy life lost and premature mortality; (2) the leading risk factors for CVD –can be diagnosed and treated at a relatively low cost;4 and (3) understanding how CVD risk varies globally could inform health systems planning and targeting of global and national CVD programs.

About 60% of all cardiovascular deaths will occur in asymptomatic people who have not had a previous event. The challenges of identifying people at high risk are significant especially in resource constrained environments with limited levels of access to services and low skilled workforces. Over the past decade, there has been a fundamental shift in the clinical paradigm for cardiovascular disease prevention, away from an approach based on defining and managing single risk factor abnormalities towards basing the need for and intensity of risk factor management based on an evaluation of a person’s future risk of experiencing a cardiovascular event. Assessment of a person’s total or absolute risk of a cardiovascular disease (CVD) event based on multiple risk factors has been demonstrated to be superior to assessment of single risk factors when identifying who is at greatest risk of a CVD event.

Objective

Taking a country level health system perspective we aim to: (1) estimate the overall and country specific CVD risk profiles and examine socio-demographic factors that contribute to variation in risk between and within countries; (2) determine unmet need for care in terms of gaps in management of people identified at elevated CVD risk based on use of guideline-recommended medications and attainment of treatment targets.

Cohort Description

We pooled datasets from nationally representative population-based surveys in LMICs. The approach to identifying and obtaining these datasets is described in the supplementary information (S11). In brief, data were obtained from the Stepwise Approach to Surveillance Surveys, and national surveys after 2005 based on a systematic review of the literature. The requirements for dataset inclusion in this study were as follows: (1) individual level data for people without prior cardiovascular disease aged over 30 years; (2) availability of all the essential variables needed to estimate CVD risk (age, sex, smoking status, systolic blood pressure and blood pressure medication use, diabetes status based on self-report or on biochemical criteria, body weight and height.

Data Sources

| **Country** | **Source** | **Year** |
| --- | --- | --- |
| Albania | Demographic and Health Survey | 2008 |
| Algeria | STEPS | 2016 |
| Azerbaijan | STEPS | 2017 |
| Belarus | STEPS | 2016 |
| Belize | Central America Diabetes Initiative | 2005-6 |
| Benin | STEPS | 2015 |
| Bhutan | STEPS | 2014 |
| Botswana | STEPS | 2014 |
| Brazil | Pesquisa Nacional de Saude(PNC) | 2013 |
| Burkina Faso | STEPS | 2013 |
| Cambodia | STEPS | 2010 |
| Chile | National Health Survey | 2009-10 |
| China | China Health and Nutrition Survey | 2009 |
| Comoros | STEPS | 2011 |
| Costa Rica | STEPS | 2010 |
| Ecuador | Ecquador National Health and Nutrition Survey | 2012 |
| Georgia | STEPS | 2016 |
| Ghana | SAGE | 2007/8 |
| Grenada | STEPS | 2009-11 |
| Guyana | STEPS | 2016 |
| India | Demographic and Health Survey | 2015-2016 |
| Indonesia | Indonesian Family Life Survey | 2014 |
| Kazakhstan | Household Health Survey | 2012 |
| Kenya | STEPS | 2015 |
| Kyrgyzstan | STEPS | 2013 |
| Lebanon | STEPS | 2008-09 |
| Lesotho | Demographic and Health Survey | 2014 |
| Liberia | STEPS | 2011 |
| Mexico | Mexican Family Life Survey | 2009-12 |
| Moldova | STEPS | 2013 |
| Mongolia | STEPS | 2009 |
| Morocco | STEPS | 2017 |
| Mozambique | STEPS | 2005 |
| Namibia | Demographic and Health Survey | 2013 |
| Nepal | STEPS | 2013 |
| Russian Federation | Study on Global Ageing and Adult health | 2007/8 |
| St. Vincent & the Grenadines | STEPS | 2013 |
| Sudan | STEPS | 2016 |
| Swaziland | STEPS | 2014 |
| Tajikistan | STEPS | 2016 |
| Tanzania | STEPS | 2012 |
| Timor Leste | STEPS | 2014 |
| Togo | STEPS | 2010 |
| Uganda | STEPS | 2014 |
| Vanuatu | STEPS | 2011 |
| Zanzibar | STEPS | 2011 |

Outcomes

**Estimation of risk:** There are multiple risk prediction equations available for the purposes of calculation of CVD risk. In this study we will use a re-calibrated version of the 2008 D’Agostino ‘low information’ Framingham risk score which calculates a 10-year CVD risk score using the variables described above. The equation is validated for people aged 30-74 years. CVD is defined as any of the following: fatal or non-fatal thromboembolic stroke, ischaemic heart disease or peripheral vascular disease. Country specific recalibrated equations were derived using CVD incidence data obtained from the 2017 Global Burden of Disease (ref). As a sensitivity analysis, we will compare the Framingham risk estimates with the 2007 World Health Organisation/ International Society of Hypertension risk charts 2007.

**Estimation of management gaps**

As one measure of management gaps we focussed on blood pressure control. Although management of all risk factors is recommended for people at elevated CVD risk there are few other variables in the dataset that can capture treatment controls. Use of other cardiovascular medicines such as blood thinning and cholesterol medications were not collected in these surveys nor were biochemical variables such as serum cholesterol levels and HBA1C for those with diabetes. Elevated CVD risk and indications for use of BP medication was based on WHO guidelines and was defined as the presence of any of the following: an extreme BP elevation (SBP >160mmHg or DBP >100mmHg); (3) a 10-year CVD risk ≥ 30%; (4) a 10-year CVD risk of 20-29% and a SBP>140 mmHg. Attainment of targets was defined as those at elevated CVD risk who were taking at least one BP lowering medication and had an SBP <140mmHg.

Statistical Methods

Because of different age profiles included in each country’s sample and because age is a major driver of risk estimates each country sample will be age standardised to the world population profile for people aged 30-74 years. The median 10-year risk for each country will then be calculated based on the risk estimates for individuals with complete data. The primary unit of analysis is the country’s health system and so consequently each risk estimate will be equally weighted. We will estimate the prevalence of the main outcomes (CVD risk and unmet needs for care) using survey analysis methods that account for the complex sampling procedure and weighting scheme. The association between the main outcomes and socio-demographic variables will be estimated using survey regression methods. Demographic and socioeconomic variables include age, sex, marital status, educational attainment, employment status, household wealth, and household income and expenditure and analyses by these groups will be presented.

Results

Table 1: Weighted distribution of CVD risk factors in 30-74 year old participants from population-based surveys.

Figure 1: CVD risk profile by country for men and women. Data missing in the overall sample and by country (S3). Supplementary Table 4 shows the difference in participants characteristics for those with and without missing data.

Figure 2: Variation in median 10-year CVD risk based by sex, level of educational status and wealth quintile.

Figure 3: Usage of blood pressure lowering medication by risk category for each country by sex.

Figure 4: Usage of blood pressure lowering medication for each country by indication and by sex

Figure 5: BP control rates by risk category and country.

Amendments

The following changes were made subsequently to the statistical analysis plan.

1. For estimation of 10-year risk of a CVD event, defined as myocardial infarction or stroke, the newly updated, non-laboratory based WHO CVD risk prediction equations were published in 2019. The non-laboratory WHO risk equation uses age, sex, smoking status, SBP, and body mass index. The equation is validated for people aged 40-80 years, we calculated risk for people aged 30-39 years assuming their age was 40 years.
2. For cross-country comparison, the median 10-year risk for each country was calculated based on the risk estimates for individuals with complete data in the age range 30-64 years with the exception of India (upper age limit 49 years for females and 54 years for males) and Ecuador (upper age limit 59 years for both males and females). Each country sample was age-standardised to the 2017 world population profile for people aged 30-64 years as per 2017 GBD estimate.
3. For people currently taking medication for high blood pressure, pre-treatment blood pressure levels were estimated using the Wald equation and used to estimate 10-year CVD risk and management gaps.
4. A sensitivity analysis was conducted where each country was weighted according to its population size in 2015.
5. Figure 3 on usage of BP lowering medication by risk category was ammended slightly to usage of BP lowering medication for those with an indication for medication.
6. BP lowering medication by 5 on BP control rates was not included in the final paper
