# Supplementary material for: Cardiovascular disease risk profile and management practices in 45 low-income and middle-income countries: A cross-sectional study of nationally representative individual-level survey data
Source: PLoS Med. 2021 Mar 4;18(3):e1003485. doi: 10.1371/journal.pmed.1003485 (PMC7932723; doi:10.1371/journal.pmed.1003485)
Supplement: S3 Text — (DOCX) [file pmed.1003485.s013.docx]

## Data access information

Data were extracted from STEPs or other similar surveys (see table S1). Methods and questionnaires used in each of these surveys can be found at the below sources:

- STEPs: https://www.who.int/ncds/surveillance/steps/instrument/en/
- DHS: https://dhsprogram.com/What-We-Do/Survey-Types/SPA.cfm
- SAGE: https://www.who.int/healthinfo/sage/en/Camdi at https://iris.paho.org/handle/10665.2/7687
- PNS, Brazil: https: //www.ibge.gov.br/en/statistics/social/justice-and-security/16840-national-survey-of-health.html?edicao=19375&t=sobre
- NHS, Chile: http://epi.minsal.cl/encuesta-ens-anteriores/
- CHNS, China: https://www.cpc.unc.edu/projects/china
- ENSANU, Ecuador: https://www.salud.gob.ec/encuesta-nacional-de-salud-y-nutricion-ensanut/.
- EHIS, Egypt: https://dhsprogram.com/pubs/pdf/FR313/FR313.pdf
- EHS, Fij: https://www.tandfonline.com/doi/full/10.3109/09286586.2011.628135
- NFHS, India http://rchiips.org/NFHS/factsheet_NFHS-4.shtml
- IFLS, Indonesia: https://www.rand.org/well-being/social-and-behavioral-policy/data/FLS/IFLS/download.html
- SANHANES, South Africa: http://www.hsrc.ac.za/en/research-areas/Research_Areas_PHHSI/sanhanes-health-and-nutrition
- HHS, Khazakstan: https://www.academypm.org/language/en/household-health-surveys/.
- MxFLS, Mexico: http://www.ennvih-mxfls.org/english/ennhiv-3.html
- SEPHAR, Romania: http://www.mymed.ro/studiul-sephar.html

**Country-specific contact information regarding accessing cascade data used in this study**

Data included in this study are only publically available for 18 of the 44 countries. The links to where data can be downloaded (upon free registration) are:

- Albania: https://dhsprogram.com/data/dataset/Albania_Standard-DHS_2008.cfm?flag=0
- Azerbaijan: https://dhsprogram.com/data/dataset/Azerbaijan_Standard-DHS_2006.cfm?flag=1
- Bangladesh: https://dhsprogram.com/data/dataset/Bangladesh_Standard-DHS_2011.cfm?flag=0
- Brazil: https://www.ibge.gov.br/estatisticas/downloads-estatisticas.html
- Chile: https://www.minsal.cl/estudios_encuestas_salud/
- China: https://www.cpc.unc.edu/projects/china/data/datasets
- Ecuador: https://ensanut.insp.mx/encuestas/ensanut2012/descargas.php
- Egypt: https://dhsprogram.com/data/dataset/Egypt_Special_2015.cfm?flag=1
- Ghana: https://apps.who.int/healthinfo/systems/surveydata/index.php/catalog/sage
- India: https://dhsprogram.com/data/dataset/India_Standard-DHS_2015.cfm?flag=0
- Indonesia: https://www.rand.org/labor/FLS/IFLS/access.html
- Kyrgyzstan: https://dhsprogram.com/data/dataset/Kyrgyz-Republic_Standard-DHS_2012.cfm?flag=1
- Lesotho: https://dhsprogram.com/data/dataset/Lesotho_Standard-DHS_2014.cfm?flag=1
- Mexico: http://www.ennvih-mxfls.org/english/ennhiv-3.html
- Namibia: https://dhsprogram.com/data/dataset/Namibia_Standard-DHS_2013.cfm?flag=0
- Peru: https://dhsprogram.com/data/dataset/Peru_Continuous-DHS_2012.cfm?flag=1
- Russia: https://apps.who.int/healthinfo/systems/surveydata/index.php/catalog/sage
- Ukraine: https://dhsprogram.com/data/dataset/Ukraine_Standard-DHS_2007.cfm?flag=1

For the remaining countries, please contact Paul Martin at pmartin@hsph.harvard.edu

* For the member countries of the Caribbean Public Health Agency (CARPHA) - Belize, Grenada, Guyana and Saint Vincent and the Grenadines - data were shared through a Data Use Agreement signed with the Executive Director of CARPHA. The Senior Technical Officer for NCDs (Dr.Glennis Andall-Brereton) should be contacted in addition to Paul Martin, if necessary.
