# Supplementary material for: Cardiovascular disease risk profile and management practices in 45 low-income and middle-income countries: A cross-sectional study of nationally representative individual-level survey data
Source: PLoS Med. 2021 Mar 4;18(3):e1003485. doi: 10.1371/journal.pmed.1003485 (PMC7932723; doi:10.1371/journal.pmed.1003485)
Supplement: Attachment — Submitted filename: Response.docx [file pmed.1003485.s014.docx]

**Requests from the editors:**

**Abstract**

1. The background section of the abstract should clearly state the aim of the study

**Response:** Amended as requested with the following text:

*“Focussing on 45 low-income and middle-income countries (LMICs), we aimed to determine i) the adult population’s median ten-year predicted CVD risk, including its variation within countries by socio-demographic characteristics, and ii) the prevalence of self-reported blood pressure (BP) medication use among those with and without an indication for such medication as per World Health Organization (WHO) guidelines”*

2. Please provide participant demographics in the abstract

**Response:** In keeping with our country level analysis, we do not think it is useful to present summary demographic statistics in the abstract. The female proportion is oversampled in India with 320,763 participants female and 57,737 males and this will unduly influence any aggregated summary statistics. For this reason, when looking at any cross country comparisons each country was equally weighted and disaggregated by sex.

3. The last sentence of the methods and findings section should outline 2-3 limitations of your study design/methodology

**Response:** The following sentence has been added to the abstract.

*“Study limitations include variation in country survey methods, most notably the sample age range and year conducted, insufficient data to use the laboratory-based risk equations, and an inability to determine past history of a CVD diagnosis”*

4. Conclusions should be tempered with “our study shows….” or similar

**Response**: Amended as requested

5. Data sharing: b) If the data are owned by a third party but freely available upon request, please note this and state the owner of the data set and contact information for data requests (web or email address). Note that a study author cannot be the contact person for the data.

**Response:** We have included the following statement in the data availability statement in the submission: *“Data cannot be shared publicly because the data use agreements prohibit this, however upon acceptance of the article, the data analysis code will be uploaded to a publicly accessible Harvard Dataverse page. We have also included in the supplementary material additional information on accessing country-specific datasets (File S11).”*

**Author summary**

1. Perhaps introduce CVD and LMIC on first view for the author summary?

**Response**: The first two dot points have been amended as follows:

- *CVD burden in low-income and middle-income countries (LMICs) is large and rising*
- *CVD risk estimation using validated risk prediction equations is recommended in most guidelines, however, there are few population representative analyses of CVD risk and its association with socio-demographic characteristics*

**Manuscript**

1. References- please place these in square brackets, placing the punctuation after the bracket

**Response:** Amended as requested

2. The section “role of the funding source” should be removed from the main text and placed in the financial information section of the article meta-data

**Response:** Amended as requested

3. Please present and organize the Discussion as follows: a short, clear summary of the article's findings; what the study adds to existing research and where and why the results may differ from previous research; strengths and limitations of the study; implications and next steps for research, clinical practice, and/or public policy; one-paragraph conclusion.

**Response**: The discussion has been reorganized as suggested.

4. Please add the following statement, or similar, to the Methods: "This study is reported as per the Strengthening the Reporting of Observational Studies in Epidemiology (STROBE) guideline (S1 Checklist)."

**Response:** This has been added in the first sentence of the methods (line p.8 16)

5. Did your study have a prospective protocol or analysis plan? Please state this (either way) early in the Methods section.

**Response:** We have added the following information in the first paragraph of the methods: *“A study outline, including proposed figures and tables, was developed prior to conducting the analyses. No changes were made to these analyses, however an initial pre-planned analysis on blood pressure control rates was excluded due to low numbers with available data”*

**Reviewer #1**

1. My major question is the degree to which this study provides new information beyond the information provided in 2019 by the WHO CVD Risk Chart Working Group (S. Kaptoge et al, 2019), as Kaptoge et al report 10-year CVD risk across 21 global regions.

**Response:** The dataset used and analyses conducted in this study go beyond the Kaptoge paper because: (1) there are several non-STEPS datasets included in the analysis whereas Kaptoge’s paper only used STEPS surveys, (2) we include important analyses of sociodemographic characteristics of participants (focussing on wealth and education); and (3) we assess use of blood pressure medication by the risk status of the individual. This has allowed us to make three novel findings:

1. A consistent inverse association between higher education, greater wealth and current employment and lower CVD risk in most countries. This underscores a need to depart from considering CVD as a disease of affluence and the importance of considering equity dimensions in identifying populations at greatest risk.
2. An underuse of medicines in people at elevated CVD risk across all countries (only 24.2% of males and 41.6% of females at high CVD risk are taking guideline-recommended BP medication).
3. A relative overuse of medicines in people at lower levels of CVD risk - 47% of all BP medication is used by people at low CVD risk without a guideline indication for its use which represents an inefficient use of scarce resources in many countries.

These novel findings are highly relevant to policy makers as they suggest who should be targeted for CVD risk factor prevention and management strategies and how these strategies should be improved to ensure that health services make efficient use of resources. Please also see the response to Reviewer 3, comment 2 for further discussion on the implications in relation to clinical guidelines.

2. Indeed, the work by Kaptoge et al highlight what I believe is a main limitation of this manuscript, namely the inferior performance of the non-laboratory risk equation. Indeed, Kaptoge describe the pragmatic models used herein as useful pre-selection tools given their poor performance among participants with diabetes. What are the implications for this finding for this manuscript, considering high global prevalence of diabetes (~9%) that shows marked heterogeneity by country?

**Response:** We agree this is an important limitation that warrants further discussion in the study implications and limitations. In the derivation cohorts in the Kaptoge paper the C-statistics for non-lab vs lab are generally quite close. See figure below

However, the reviewer rightly points out that the laboratory-based equations tend to outperform non-laboratory equations for people with diabetes, with the non-laboratory equations tending to underestimate risk. The implications for our study particularly pertain to people with diabetes who may be assessed at low risk but may be re-classified to be at higher risk using the laboratory-based equation. For people in this group taking BP medication, we may have overestimated the proportion taking medication without a clear indication. Conversely for people in this group not taking medication, it would lead to an underestimation of the treatment gap.

We have added the following text in the methods section:

*In almost all survey samples, cholesterol data were not available and in many country survey samples diabetes status was also not available. Consequently, the non-laboratory WHO risk equation was the more appropriate equation to use given it does not rely on either of these variables. Although it discriminates similarly to laboratory equations overall, it is known to underestimate risk in people with diabetes.[16]*

And the following text in the limitations section

*“Fifth, use of the non-laboratory equations may mean that some people, particularly those with diabetes, who were assessed at low risk may be re-classified to be at higher risk if laboratory values were available. This could lead to an overestimate in the proportion found to be taking BP medication without a clear indication and conversely for those not on BP medication, it could lead to an underestimate of the treatment gap.”*

Despite this important limitation, in many countries laboratory cholesterol values are not routinely ordered and assessment of risk is dependent on non-laboratory equations. Also given STEPS and other surveys generally do not routinely collected laboratory measures, non-laboratory estimates are generally the only means by which population-representative risk estimates can be obtained. This sentence has also been added to the text in the same section.

3. The decision to include participants <40 years of age also was poorly motivated and potentially erroneous, particularly since the equations are not calibrated for this age-group. It is therefore difficult to know how inclusion of these individuals, who are likely at very low CVD risk, affected the results, although one can speculate that they shifted countrywide estimates of 10-year CVD risk downwards.

**Response:** In assessing risk for those less than 40 years we calculated their risk at age 40 years. A key reason for including people aged 30-39 years is that overall 9.4% (interquartile range 4.2 – 14.3) of people taking BP medication are in this age range, with the majority having pre-treatment risk levels suggesting no clear indication for taking medication. Please also see the response to reviewer 3, comment 1 on why we feel this is important.

We have conducted a sensitivity analysis to show the risk profile, excluding those aged 30-39 years and added this to the supporting information (S5). We have added the following sentence to the results:

*"In a sensitivity analysis, risk was calculated excluding those under 40 years of age and median (IQR) risk increased to 4.0% (3.4 - 5.9) for males and to 2.8% (2.2 - 3.4) for females (S5)."*

4. I also agree with the authors when then speculate that the inability to exclude prevalent cases likely biased their results downward. Yet, the prevalence of CVD likely varies across countries, complicating country-specific comparisons. The degree to which inclusion of prevalent cases affects cross-sectional associations reported in Figure 2 also is difficult to ascertain.

**Response:** In taking a country level perspective in this paper, the primary purpose was to examine within country risk profiles, sociodemographic variation by risk and use of BP medication by risk status. The heterogeneity across countries at every level (sociodemographic, risk factor profiles, use of BP meds) precludes meaningful cross-country comparisons to be made and we have generally avoided such analyses.

In relation to Figure 2, if CVD prevalence exhibits sociodemographic variation in the opposite direction to the associations we observed this could lead to a bias away from the null. Conversely if the variation is in the same direction then the association may be stronger than observed. However, this is speculative and we cannot make any definitive comment about the implications.

5. Together, the limitations described above affect efforts to ascertain medicine overuse and underuse vis-à-vis anti-hypertensive agents.  Limitations of the risk score aside, how much of the apparent discrepancy between medication usage and risk factor profile is captured by measurement error in blood pressure? These limitations call into question the authors statement that "the majority of BP medication is being used by people at lower levels of CVD risk."

**Response:** The STEPS and other surveys use standardised processes for measurement of blood pressure and other physical examination procedures to minimise measurement error and therefore we do not think this would be a major concern in this study.

6. Minor comments include conflation of CVD incidence and CVD mortality in the introduction as well as statements suggesting that leading risk factors for CVD can be diagnosed and treated at relatively low cost. (This statement ignores the rising tides of diabetes and obesity.)

**Response**: Thanks, we have amended the first sentence to refer to disease burden (not just mortality). By relatively low cost we mean that risk factors for CVD can be treated at low cost, relative to the costs of treating CVD events such as hospitalisation for myocardial infarction and stroke. We have added this clarification in the introduction.

7. Finally, this paper seems to take a "prevention by treatment of high-risk individuals approach" as opposed to more population-wide approaches advocated by Geoffrey Rose, among others. The degree to which targeting high-risk individuals in resource strained settings remains unclear.

**Response:** It is true that this paper is focussed on clinical management of people according to cardiovascular risk status as recommended by WHO guidelines and the focus is restricted to looking at guideline-practice gaps. Despite the importance of treating high risk individuals, population-wide approaches are critically important and we most certainly do not suggest these should be neglected as a strategy to shift whole populations toward lower CVD risk. We have added this sentence in the implications paragraph of the discussion to emphasise this point.

*“The case for investing in CVD prevention is therefore greater than ever. The WHO estimated that a US $120 billion investment in 20 high-burden countries ($1.50 per capita over 15 years) would avert 15 million deaths and 21 million incident ischaemic heart disease or stroke incidents in these countries [4].* ***This includes investing in population-level strategies to lower CVD risk and strategies to improve health care performance”***

**Reviewer #2:**

I confine my remarks to statistical aspects of this paper.  The general method is fine, but I have some issues and questions to resolve before I can recommend publication.

1. p. 7 Line 3 “Leading cause" is almost meaningless as it depends on how causes are defined. Give rates of death and disability.

Response: The first sentence of the introduction has been amended as follows:

***“****Although cardiovascular disease (CVD) disease burden is declining in high-income countries, it is rising in low-income and middle-income countries (LMICs) and is the leading cause of death worldwide, accounting for an estimated 17.8 million deaths and an age-standardised death rate of 233 per 100,000 in 2017 [1]*.”

2. p. 11 line 8 Do you mean "simple linear regression"?  (That is, one IV and one DV)? It's also not clear what you mean by "primary” --- do you mean "initial"? "Preliminary?"   If you mean a process of bivariate screening, that is not a good method of building a model.

**Response:** Thanks, we have deleted this sentence as it is a little confusing. All the analyses presented are multiple variable models.

3. line 11 "multivariate" should be "multiple"

**Response:** Amended as suggested

4. line 12, 13 What covariance pattern was used?

**Response:** A compound symmetry or exchangeable covariance structure was used to model correlation between individuals belonging to the same primary sampling unit (cluster).

5. Line 15 Don’t use quintiles and, if you have to use quintiles, don't combine them. The best would be to use an index.  Categorizing continuous IVs is almost always a mistake. In *Regression Modelling Strategies* Frank Harrell lists 11 problems with this and sums up "nothing could be more disastrous".

**Response:** We combined quintiles while performing the multiple regression so that we were able to fit a regression model for countries with few data points. We agree that categorizing continuous variables is, in general, a bad idea. Also, fitting a wealth index as a continuous variable would require fewer parameters. However, wealth quintiles are familiar and useful concepts in public health and social determinants of health literature and are used in policy planning in many countries. If we were to generate a wealth index as the continuous independent variable, we may want to include a squared or a cubic term to incorporate a non-linear relationship between household wealth and logarithm of 10-year CVD risk. While this is a more efficient use of the information in the variable, interpretation of the regression coefficients is more difficult. We are happy to take editorial advice on this issue.

6. line 17  Why was Poisson regression used? For dichotomous outcome variables, the usual choice is logistic regression

**Response:** For binary outcome variables, the parameter of most interest to us to assess the risk of exposure is relative risk. Logistic regression will yield an odds ratio which closely approximates relative risk when the outcome is rare. However, given in our data the rare event assumption does not hold, an odds ratio may not correctly approximate to relative risk. For this reason, we used a modified Poisson regression approach (i.e., Poisson regression with a robust error variance) to generate a relative risk estimate.

7. p. 12 line 20   "increased" is too causal a term. You can use "was higher" or something similar

**Response:** Amended as suggested

8. Figure 1 - stacked bar plots are not a good method. See the work of William S., Cleveland.  Here, I think density plots would be better (and that avoids categorizing the risk)

**Response:** Thanks for raising this point. It is a tricky issue to deal with because we agree that density plots reveal more information about continuous variables and a ridgeline plot may be a better alternative to compare risk distribution across countries. The counter-argument is that CVD risk categorisation is essentially what is used to drive clinical decision making and is recommended in guidelines. On balance, we felt that this makes interpretation easier for clinicians. Additionally, a stacked bar graph makes it a little easier to visualise the proportion of men and women in each category across various countries. We are happy to be guided by the editors on what presentation is most suitable for Plos Med readers.

9. Figure 2 - The axes for the different variables should all be the same scale F and wealth should not be categorized.

**Response:** Amended as suggested – please see revised figure. See above point regarding wealth categorisation.

10. Figure 3 is a "dynamite plot" These are not recommended. A Cleveland dot plot may be better

**Response:** Amended as suggested – please see revised figure. We have also included the 95% confidence intervals on the risk ratios.

**Reviewer #3:**

1. The authors rationale for the study is based on a number of assumptions that have implications on the inevitable findings, but for which little evidence is provided to support.  The first is that WHO PEN is the most widely used technical guidance document in LMICS and therefore the guidance that most influences primary care practice. Unfortunately, we are not provided with any evidence that this is true. The evidence that is provided by the authors (references) is that the PEN guidelines are feasible but not very effective where used (in part because the uptake was poor).
2. The second assumption is that most countries and health systems use the WHO PEN or GLOBAL HEARTS approach, which recommend CVD risk estimation-for decisions on treatment interventions for interventions.  Many (perhaps most) countries across sub Saharan Africa, Indian sub-Continent Asia, Middle East and South America also have local or regional Blood Pressure Management guidelines and or access to WHO and International Society of Hypertension (or combined) guidelines on the management of blood pressure.  In contrast to the WHO PEN guidance, almost all of these blood pressure guideline documents dating back to the early 2000s stipulate that if "low risk" hypertensive patients do not respond to lifestyle interventions (diet, weight loss, exercise) within 3-6 months, they should be treated with an antihypertensive agent. This is important because if it is true that most of the globe integrates separate hypertension guidelines to management in their primary health care approaches, the research question may be somewhat rhetorical i.e., at attempt to prove what we "know" rather than find out what we do not know. There is also a distinct possibility that the reason for one of their main findings -that low risk patients are "overtreated" may be by design-i.e., because the health system and health care practitioners are adhering in part to local guidance on BP treatment and not lack of adherence to WHO guidance. The 47% of people on BP treatment who they find do not have an indication for BP may infect have a clear indication based on current and former hypertension guidelines.

**Response:** The reviewer raises a critical point that is central to the paper - namely to what extent should ‘best practice’ be defined by these particular guidelines. The second comment is equally pertinent and relates to the use of local and single risk factor-oriented hypertension guidelines as potentially more relevant benchmarks for guiding best practice. This further emphasises the novelty and importance of this study and the need for greater discussion on this issue.

The fundamental premise of our paper is that risk-based management is superior to individual risk factor management and this will lead to better allocation of resources for a population that is likely to gain the most. We have cited papers to support the benefits of a risk based approach starting with the seminal paper by Jackson et al in the Lancet in 2005 (reference 14) and a more recent paper published in Plos Medicine in 2018 (Karmali Reference 22) which showed specific to BP lowering treatment that a risk based approach was superior (area under the curve 0.71 [95% confidence interval (CI) 0.70–0.72] for a CVD risk strategy versus 0.54 [95% CI 0.53–0.55] for a single risk factor strategy).

We agree wholeheartedly that what we have observed is persistence of the single risk factor paradigm to a large extent. These individuals may well be indicated for BP treatment based on hypertension guidelines despite not being recommended based on risk. We do not think we have emphasised the importance of this finding. Rather than this being a study about following guidelines, it may be about following guidelines that potentially are conflicting.

We have drawn on the reviewer’s comments and added the following sentence in the discussion:

*“An important explanation for this pattern is the likely persistence of single risk factor-based treatment over risk-based treatment. Many countries have local or regional hypertension guidelines and these guidelines which have been in operation for decades generally recommend that if hypertensive patients do not respond to lifestyle interventions (diet, weight loss, exercise) within 3-6 months, they should be treated with an antihypertensive agent. Given the superiority of risk-based management, there needs to be harmonisation of conflicting guidelines and a shift toward targeting treatment in individuals who will gain the greatest benefit in terms of CVD events avoided.*

3. The methods and analytical approach to address the questions are appropriate.  The results and their interpretation are sound. The only question is that one may ask is whether the study has addressed any significant gaps in knowledge or strengthened the available evidence

**Response:** Please see the response to reviewer 1, comment 1
